# The Canadian prospective cohort study to understand progression in multiple sclerosis (CanProCo): rationale, aims, and study design

**DOI:** 10.1186/s12883-021-02447-7

**Published:** 2021-10-27

**Authors:** Jiwon Oh, Nathalie Arbour, Fabrizio Giuliani, Melanie Guenette, Shannon Kolind, Larry Lynd, Ruth Ann Marrie, Luanne M. Metz, Scott B. Patten, Alexandre Prat, Alice Schabas, Penelope Smyth, Roger Tam, Anthony Traboulsee, V. Wee Yong

**Affiliations:** 1 Division of Neurology, St. Michael’s Hospital, University of Toronto, 30 Bond Street, Toronto, ON M5B 1W8 Canada; 2 Department of Neurosciences, Université de Montréal and Centre hospitalier de l’Université de Montréal, 900 rue St. Denis, Montreal, QC H2X 0A9 Canada; 3 Division of Neurology, Department of Medicine and Neuroscience and Mental Health Institute, University of Alberta, 11350-83 Avenue, Edmonton, AB T6G 2G3 Canada; 4 Department of Medicine, Division of Neurology, University of British Columbia, 2221 Wesbrook Mall, Vancouver, BC V6T 2B5 Canada; 5 Department of Radiology, University of British Columbia, 2221 Wesbrook Mall, Vancouver, BC V6T 2B5 Canada; 6 Faculty of Pharmaceutical Sciences, University of British Columbia, 2405 Wesbrook Mall, Vancouver, BC V6T 1Z3 Canada; 7 Centre for Health Evaluation and Outcome Sciences, Providence Health Research Institute, 1081 Burrard Street, Vancouver, BC V6Z 1Y6 Canada; 8 Departments of Internal Medicine and Community Health Sciences, Max Rady College of Medicine, Rady Faculty of Health Sciences, University of Manitoba, 744 Bannatyne Ave, Winnipeg, MB R3E 0W2 Canada; 9 Department of Clinical Neurosciences, University of Calgary Foothills Hospital, 1403-29th Street NW, Calgary, AB T2N 2T9 Canada; 10 Department of Community Health Sciences, University of Calgary, 3280 Hospital Drive NW, Calgary, AB T2N 4Z6 Canada; 11 School of Biomedical Engineering, University of British Columbia, 2222 Health Sciences Mall, Vancouver, BC V6T 1Z3 Canada; 12 Department of Clinical Neurosciences and the Hotchkiss Brain Institute, University of Calgary, 2500 University Drive NW, Calgary, AB T2N 1N4 Canada

**Keywords:** Multiple sclerosis, Cohort, Prospective, Progression, Progressive MS, Epidemiology, Imaging, Neuroimmunology, Biology, Health systems, Canada

## Abstract

**Background:**

Neurological disability progression occurs across the spectrum of people living with multiple sclerosis (MS). Although there are a handful of disease-modifying treatments approved for use in progressive phenotypes of MS, there are no treatments that substantially modify the course of clinical progression in MS. Characterizing the determinants of clinical progression can inform the development of novel therapeutic agents and treatment approaches that target progression in MS, which is one of the greatest unmet needs in clinical practice. Canada, having one of the world’s highest rates of MS and a publicly-funded health care system, represents an optimal country to achieve in-depth analysis of progression. Accordingly, the overarching aim of the Canadian Prospective Cohort Study to Understand Progression in MS (CanProCo) is to evaluate a wide spectrum of factors associated with the clinical *onset* and *rate* of disease progression in MS, and to describe how these factors relate to one another to influence progression.

**Methods:**

CanProCo is a prospective, observational cohort study with investigators specializing in epidemiology, neuroimaging, neuroimmunology, health services research and health economics. CanProCo’s study design was approved by an international review panel, comprised of content experts and key stakeholders. One thousand individuals with radiologically-isolated syndrome, relapsing-remitting MS, and primary-progressive MS within 10–15 years of disease onset will be recruited from 5 academic MS centres in Canada. Participants will undergo detailed clinical evaluation annually over 5 years (including advanced, app-based clinical data collection). In a subset of participants within 5–10 years of disease onset (*n* = 500), blood, cerebrospinal fluid, and research MRIs will be collected allowing an integrated, in-depth evaluation of factors contributing to progression in MS from multiple perspectives. Factors of interest range from biological measures (e.g. single-cell RNA-sequencing), MRI-based microstructural assessment, participant characteristics (self-reported, performance-based, clinician-assessed, health-system based), and micro and macro-environmental factors.

**Discussion:**

Halting the progression of MS remains a fundamental need to improve the lives of people living with MS. Achieving this requires leveraging transdisciplinary approaches to better characterize why clinical progression occurs. CanProCo is a pioneering multi-dimensional cohort study aiming to characterize these determinants to inform the development and implementation of efficacious and effective interventions.

**Supplementary Information:**

The online version contains supplementary material available at 10.1186/s12883-021-02447-7.

## Background

Multiple sclerosis (MS) is an immune-mediated disease of the central nervous system and one of the most common chronic neurological disorders in young adults in Canada and many other parts of the world. In 2021, nearly 100,000 Canadians were affected by MS, which has substantial personal, professional, and societal consequences [1].

There has been considerable progress in the field of MS, including the development of many treatment options for the relapsing-remitting (RRMS) phenotype of the disease. However, there are few treatments for progression in MS (i.e. primary progressive [PPMS], and secondary progressive MS) and the available treatments demonstrate only modest efficacy and are unable to substantially change the trajectory of disease progression. The lack of effective treatments for progression in MS is largely due to an incomplete understanding of the mechanisms and factors underlying disease progression.

Traditionally, MS has been divided into specific phenotypes, including relapsing remitting and progressive forms. Accumulating evidence suggests that despite the designation of MS into phenotypes of relapsing or progressive form, MS likely exists on a disease continuum and biological and clinical disease progression is a component of the disease from the onset [2]. A variety of clinical, biological, genetic, environmental, and health systems factors interact in complex ways to affect MS disease onset and clinical manifestations, which include relapses and disability progression over time. However, the specific contribution of individual factors and their interplay remain poorly understood. A thorough understanding of biological mechanisms and the complex interplay of various factors related to disease progression is a necessary stepping-stone to develop more targeted and effective disease-modifying treatments and treatment strategies for progression in MS.

In recognition of this knowledge gap, international efforts are being made to address this unmet clinical need [3]. One of Canada’s major efforts is through the establishment of The Canadian Prospective Cohort (CanProCo) Study to Understand Progression in Multiple Sclerosis, a national effort fueled by MS clinicians, scientists, and funders comprising the Canadian MS community. CanProCo is a pioneering multi-dimensional national cohort study specifically designed with the goal of better understanding the multiplicity of factors driving progression in MS over time.

CanProCo will follow individuals living with different phenotypes and stages of MS severity and progression over 5 years, collecting a wide range of clinical, biological, imaging, and health administrative information. Through careful selection of study participants, objectives, and study design (outlined in detail below), CanProCo investigators hope to answer specific questions related to how progression occurs, which we expect will provide insight into what causes the clinical onset and variable magnitude of progression across the spectrum of people living with MS. These insights will pave the way for better treatment and management strategies to prevent the accumulation of neurological disability, ultimately improving the lives of people with MS in Canada and around the world.

## Methods

This protocol adheres to the STROBE guidelines [4].

### Aim, design, and setting of the study

The over-arching goal of CanProCo is to evaluate a spectrum of factors (e.g., clinical, biological, genetic, environmental, and health systems factors) associated with the de novo clinical onset and rate of MS disease progression over a 5-year period, and to assess how these factors relate to one another.

The CanProCo study is comprised of three scientific pillars with the following specific aims:

1. *Neuro-immunology*

Identify biological factors (e.g., immune-related, metabolic, proteolytic, pro-oxidative stress and growth factor profiles) associated with:

- ▪ Absence or presence of progression along the MS disease spectrum (i.e. radiologically isolated syndrome (RIS) to RRMS, and RIS to PPMS)
- ▪ De novo clinical onset of progression in MS (i.e. progression observed in RRMS, and RIS to PPMS)
- ▪ Rate of progression (i.e. in RRMS and PPMS)

2. *Neuro-imaging*

Identify microstructural substrates in the whole brain and spinal cord, and in specific regions of the central nervous system associated with de novo clinical onset and rate of progression in MS.

3. *Epidemiology and health outcomes*

Identify and evaluate patient-level (demographic and clinical), environmental, and health system-level factors related to the de novo clinical onset and *rate* of progression in MS, and the health and health economic impact of progression.

### Integrative and exploratory aims

While each of CanProCo pillar’s specific aims are important independently, a unique strength of this study will be the integration across scientific silos by the evaluation of integrative aims, including characterizing the interactions and associations between factors in epidemiology and health outcomes, neuroimaging, and neuro-immunology associated with de novo clinical onset *and rate* of progression in MS. Exploratory aims involve exploratory use of novel data collection and analysis strategies to improve the assessment of progression in people with MS, including the possibility of predicting future de novo clinical onset *and rate* of progression in MS.

### Study sites

Over the course of a 10 month feasibility and planning grant (July 2017 – May 2018), CanProCo investigators deliberated regarding numerous logistical and methodological issues, and landed on a final study design that was thought to have the greatest potential to meet the study objectives over a 5-year period. To determine study sites, an open call was issued to Canadian MS clinics in September 2017 and potential study sites were assessed for fulfillment of minimum criteria, which included: the ability, interest, and infrastructure to contribute to all scientific pillars, availability of a neurologist to act as site clinical investigator, clinic population ≥ 1000 MS patients, and current use of an electronic MS database. Study sites were ultimately determined by interest, feasibility, and budgetary restraints.

The CanProCo study includes the following five sites: St. Michael’s Hospital at the University of Toronto (Toronto, Ontario), Centre hospitalier de l’Université de Montréal (Montreal, Quebec), Djavad Mowafaghian Centre for Brain Health at the University of British Columbia (Vancouver, British Columbia), Calgary Multiple Sclerosis Clinic (Calgary, Alberta), and Northern Alberta MS Clinic at the University of Alberta (Edmonton, Alberta).

### Recruitment and screening of participants

**Fig. 1:**
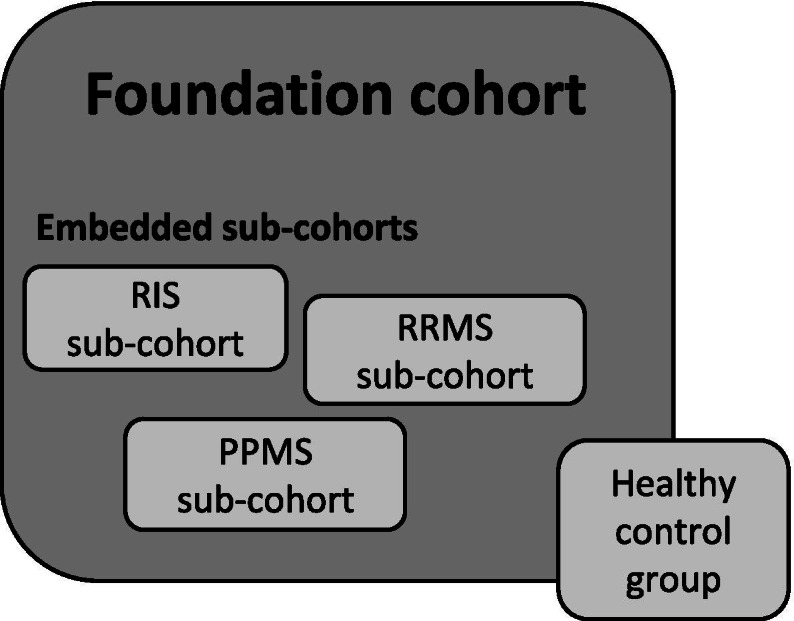
Foundation cohort with embedded sub-cohorts and healthy control group

Each CanProCo study site enrolls individuals into a base foundation cohort on whom detailed clinical data and routine clinical magnetic resonance imaging (MRI) scans of the brain and spinal cord are collected. The foundation cohort is enriched with targeted sub-cohorts (Fig. 1) and a healthy control group on whom biological samples and advanced research MRI scans of the brain and cervical spinal cord are collected. As the sub-cohorts undergo a *deep-dive* of data collection, they have therefore been designed to answer questions related to the mechanisms and impact of progression in MS.

Participants are recruited by MS clinic neurologists or other providers in the circle of care, as well as through advertisement (e.g. waiting room posters, MS Society of Canada website, Brain Canada website and social media avenues). Potential participants are referred to the study team by their neurologist (for example), or directly by self-referral via advertisement.

When recruitment is performed by the clinical care team, patients who meet study inclusion and exclusion criteria are approached. This strategy was employed to ensure an adequate sample size in several key groups to support the scientific aims of the study and was determined to be feasible during the planning grant period, when a landscape assessment was performed of Canadian MS patients at a few large MS centres with existing retrospective data.

All potential participants are screened prior to enrollment. Inclusion and exclusion criteria for the foundation cohort (participants with RIS, RRMS, PPMS within specified disease durations), embedded sub-cohorts (participants with RIS, RRMS, PPMS within specified disease durations), and healthy controls are outlined in Tables 1, 2, 3, 4, and 5. Inclusion and exclusion criteria for the foundation and embedded sub-cohorts were carefully selected to represent the optimal participant population that would allow a better understanding of factors related to the onset and rate of progression in MS over 5 years, while attempting to minimize practical issues that can affect prospective studies, including confounding factors, difficulties attending annual study visits, and attrition. Specifically, RIS participants were chosen for inclusion as they represent participants who have not yet developed overt clinical symptoms of MS, thus allowing for an evaluation of factors related to the clinical onset of MS (relapsing or progressive MS), and evaluation of progression in MS (in RIS subjects who develop PPMS). Early RRMS participants were chosen for inclusion as progression likely occurs across the spectrum of MS [2, 5], and these participants will allow for an evaluation of the onset of progression, and rate of progression in group of people with early relapsing MS, enabling an assessment of early biological, microstructural and clinical changes which are likely less confounded by external factors in comparison to those with more advanced MS. Moreover, long term follow-up the RIS and early RRMS groups will be expected to demonstrate changes in status, that will ultimately provide coverage across the full continuum of the disease when coupled with PPMS. Finally, PPMS participants were chosen as they will enable an evaluation of factors relevant to the rate of progression without superimposed relapses, in a known progressive subtype of MS.

**Table 1:** Foundation cohort inclusion and exclusion criteria

Foundation cohort (***n*** = 1000)	
**Inclusion criteria**	
Age 18–60	
EDSS [6] ≤ 6.5	
RIS diagnosis meeting Okuda [7] criteria	
RRMS diagnosis or CIS diagnosis with MRI evidence of dissemination in space [8], with symptom onset ≤10 years	
PPMS diagnosis [8] with symptom onset ≤15 years	
**Exclusion criteria**	
HIV positive	
Previous or current treatment with chemotherapy for malignancy	

*EDSS* Expanded Disability Status Scale, *RIS* Radiologically Isolated Syndrome, *RRMS* Relapsing Remitting Multiple Sclerosis, *CIS* Clinically Isolated Syndrome, *MRI* Magnetic Resonance Imaging, *PPMS* Primary Progressive Multiple Sclerosis, *HIV* Human Immunodeficiency Virus

**Table 2:** RRMS sub-cohort inclusion and exclusion criteria

RRMS sub-cohort (***n*** = 200)	
**Inclusion criteria**	
Age 18–60	
EDSS [6] ≤ 6.5	
RRMS diagnosis or CIS diagnosis with MRI evidence of dissemination in space [8], with symptom onset ≤5 years	
Treatment naïve or no disease modifying treatment ≥6 months	
At least 100 participants demonstrating “high” disease activity defined by: • 2 or more relapses in the past year OR • 1 relapse in the past year and > 10 T2 lesions and > 3 Gad+ lesion OR • 1 relapse in the past year and > 10 T2 lesions and > 3 new T2 lesions in the past 1–2 years OR • 1 relapse in the past year and > 10 T2 lesions and brainstem/spinal cord involvement (clinically or on MRI) • 1 relapse in the past year with > 10 T2 lesions with incomplete relapse recovery and EDSS > 2.0	
**Exclusion criteria**	
HIV positive	
Previous or current treatment with chemotherapy for malignancy	

N.B. Individuals having previously received induction therapies (e.g. cladribine tablets) with continuous effects or stem cell transplantations are considered on treatment and therefore not eligible for the RRMS sub-cohort. Enrolment is permitted before such a therapy is administered

*EDSS* Expanded Disability Status Scale, *RRMS* Relapsing Remitting Multiple Sclerosis, *CIS* Clinically Isolated Syndrome, *MRI* Magnetic Resonance Imaging, *HIV* Human Immunodeficiency Virus

**Table 3:** PPMS sub-cohort inclusion and exclusion criteria

PPMS sub-cohort (***n*** = 100)	
**Inclusion criteria**	
Age 18–60	
EDSS [6] ≤ 6.5	
PPMS diagnosis [8] with symptom onset ≤10 years	
**Exclusion criteria**	
HIV positive	
Previous or current treatment with chemotherapy for malignancy	

*EDSS* Expanded Disability Status Scale, *PPMS* Primary Progressive Multiple Sclerosis, *HIV* Human Immunodeficiency Virus

**Table 4:** RIS sub-cohort inclusion and exclusion criteria

RIS sub-cohort (***n*** = 150)	
**Inclusion criteria**	
Age 18–60	
EDSS [6] ≤ 6.5	
RIS diagnosis meeting Okuda [7] criteria	
**Exclusion criteria**	
HIV positive	
Previous or current treatment with chemotherapy for malignancy	

*EDSS* Expanded Disability Status Scale, *RIS* Radiologically Isolated Syndrome, *HIV* Human Immunodeficiency Virus

**Table 5:** Healthy control group inclusion and exclusion criteria

Healthy controls (***n*** = 50)	
**Inclusion criteria**	
Age 18–60	
**Exclusion criteria**	
HIV positive	
Previous or current treatment with chemotherapy for malignancy	
Previous traumatic brain injury, brain surgery, recent cancer treatment, dementia, stroke, or neurological or psychiatric (e.g., major depressive disorder) disease causing functional limitation.	

*HIV* Human Immunodeficiency Virus

Of note, one of the inclusion criteria for participants in the RRMS subcohort is that they are treatment-naïve of have not been on DMT for > 6 months. Initiation of a necessary DMT is not delayed by the study visit, as a participant is only recruited for this subcohort if the study visit can be arranged before they initiate DMT, since it would be unethical to delay initiation of DMT for recruitment purposes.

### Data collection

Standard operating procedures (SOPs) were developed and refined for each CanProCo scientific pillar during the planning period. These finalized SOPs were circulated to participating sites prior to study initiation and included detailed instructions on clinical data collection (i.e. from participant medical chart and during clinical interview), biological specimen (i.e. blood and cerebrospinal fluid (CSF)) collection, processing, and shipment, and clinical- and research-grade MRI protocols.

A face-to-face study initiation meeting was held in January 2019 and included scientific pillar leaders, individual site clinical and laboratory investigators, and research staff. Ongoing distance training continued virtually leading up to study initiation and has continued periodically as needed, specifically for research coordinators and laboratory staff.

Study visits are conducted by the CanProCo study coordinator in a dedicated clinical or research area, under the supervision of the local clinical investigator. Visits are conducted every 12 months, however given the impact of the coronavirus-19 pandemic, the initial follow-up visit is permitted to be delayed a maximum of 6 months.

Study participants retain their regular clinical follow up and care programs with their treating neurologist throughout the duration of their CanProCo enrolment.

**Fig. 2:**
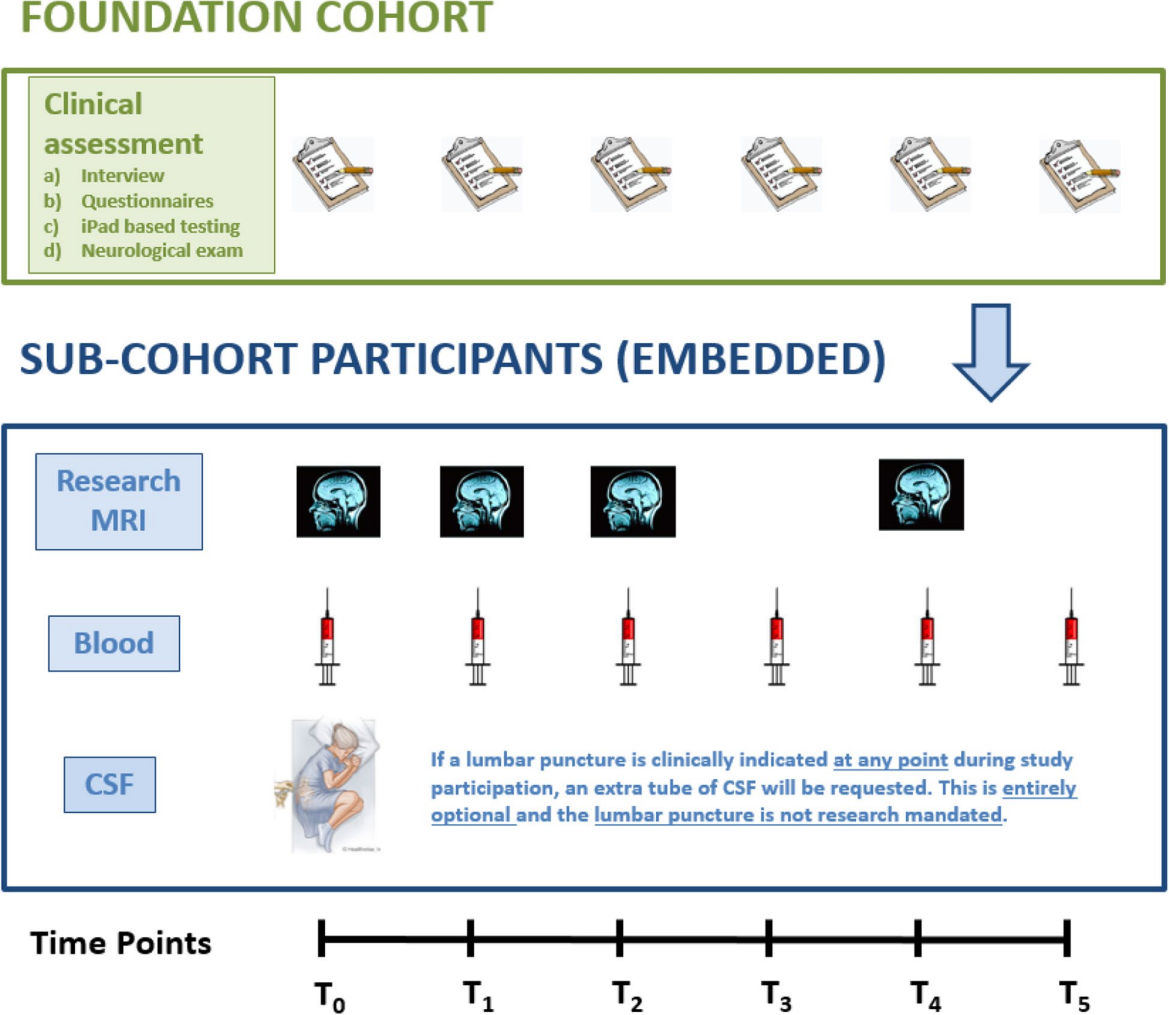
Outline of CanProCo data collection in foundation cohort and embedded sub-cohorts. This is an original figure that is owned by CanProCo Investigators

CanProCo foundation participants contribute a detailed complement of clinical and epidemiological data (i.e. questionnaires/surveys, case report forms, iPad based testing, smartphone app-based clinical data collection, and neurological exam). Embedded sub-cohort participants provide the same data, in addition to biological samples (i.e. blood, and when performed for clinical purposes, CSF) and research grade MRI scans of the brain and cervical spinal cord, which include numerous advanced sequences (Fig. 2). Healthy control participants are recruited from amongst family members of study participants, hospital staff, or by advertisement in MS clinics. Healthy control participants contribute questionnaire/survey and case report form data, in addition to blood samples and research grade MRI scans of the brain and cervical spinal cord.

#### Clinical interview

##### Case report form

All participants report basic demographic details and clinical information (Table 6) related to MS. MS symptom type and onset, and treatment history are abstracted from foundation and sub-cohort participants’ medical charts by the study coordinator for screening purposes using a standardized abstraction form and confirmed during the study visit.

**Table 6:** CanProCo case report form data

Data collection item	
Month and year of birth	
Sex and gender	
Handedness	
Country and city of birth, ethnicity	
Number of years of education	
Employment status	
Insurance provider type (i.e. public, private, combination)	
Current living arrangement (e.g. home, assisted living)	
Postal code (partial)	
Marital status and number of children	
Vitamin intake (multi-vitamin, and vitamin D)	
Alcohol intake	
Patient Determined Disease Steps [9]	
Family history of MS	
Current diagnosis	
Year of MS disease onset and diagnosis	
Onset symptoms	
Relapse and steroid treatment history	
Current and previous MS treatments	
Recent (within previous year) vaccination use and type	
Height and weight	
History of confirmed coronavirus-19 infection^a^	

*MS* Multiple Sclerosis

^a^added after study initiation

##### Questionnaires and surveys

Baseline and follow-up study visits include a standard battery (Table 7) of questionnaires and scales to measure healthcare resource utilization, quality of life, work productivity, fatigue, mood (anxiety and depression), substance use, physical activity, and comorbidities. Individual questionnaires were chosen by the epidemiology pillar leads based on the following factors: relevance to study objectives, validation in people with MS, experience with use, ease of administration, and barriers to use.

**Table 7:** CanProCo questionnaires and surveys

Questionnaire/survey name	Variable or factor of interest
Healthcare Resource Utilization Questionnaire [10]	Healthcare resource use
Euro Quality of Life – 5 Dimensions [11, 12]	Quality of life
^a^Multiple Sclerosis Quality of Life [13]	Quality of life
Health Related Productivity Questionnaire [14]	Work productivity
Valuation of Lost Productivity [15]	Work productivity
Modified Fatigue Impact Scale [16, 17]	Degree of fatigue
Patient Health Questionnaire [18]	Anxiety and depression
Generalized Anxiety Disorder [19]	Anxiety and depression
Statistics Canada Demographic Questions [20]	Tobacco, cannabis use, physical activity
Comorbidity Questionnaire [21]	Presence of concurrent medical conditions

^a^Control participants do not complete the Multiple Sclerosis Quality of Life questionnaire

Participants choose to complete questionnaires and surveys on paper copies during in-person study visits, or online using a direct entry option via the CanProCo study electronic database (Praxis Spinal Cord Institute’s Global Research Platform (GRP)).

##### Clinical assessments

**Neurological exam**

An Expanded Disability Status Scale (EDSS) [6] score is obtained via neurological exam during each dedicated research visit. A clinical EDSS done within 30 days of a research visit may be used in lieu of a research evaluation if the participant does not endorse any clinical change in the interval. An EDSS must be administered by a neuro-status [6] experienced physician.

**Multiple Sclerosis Performance Test**

The Multiple Sclerosis Performance Test (MSPT) is an iPad-based system that collects demographic and clinical data in a standardized manner [22, 23], and is an electronic method of administering a modified version of the Multiple Sclerosis Functional Composite (MSFC) [24]. The application allows study foundation and sub-cohort participants to self-administer (under the supervision of the research coordinator) tests of visual acuity, cognition, ambulation, manual dexterity, demographics, health history, and quality of life during annual study visits. Healthy control participants do not complete the MSPT. A complete list of MSPT modules is listed in Table 8.

**Table 8:** Multiple Sclerosis Performance Test (MSPT) modules

MSPT module	
MyHealth module (i.e. demographic and health history details)	
Quality of life in neurological disorders	
Contrast sensitivity test, based on Sloan Letter Chart	
Manual dexterity test, based on 9-Hole Peg Test	
Walking speed test, based on Timed 25 Foot Walk	
Information processing speed test, based on the Symbol Digit Modalities Test	

**Smartphone-based digital data collection**

Innovative mobile devices, including (Floodlight) [25], a smart-phone based application designed for people with MS will be utilized to enrich clinical data collection and to explore novel ways to monitor disease progression in MS. This component of data collection is entirely optional. Data elements that will be collected from Floodlight are outlined in Table 9 below. The Floodlight application utilizes smart-phone based tasks and questionnaires to collect a rich dataset of information on upper limb function, ambulation, cognition, mood, quality of life, and passive movement in MS patients that can be collected at suggested time points ranging from daily to weekly, between annual visits. This strategy minimizes the cost of data collection and allows for more frequent measurements in patients, which is highly relevant to a chronic disease like MS.

**Table 9:** Components of Floodlight smartphone application

Assessments/Data collected	Floodlight Open
Mood	• Daily mood questionnaire
Visual dysfunction	None
Hand-motor function	• Draw a shape • Pinching test • Thumb strength test
Gait and posture	• 2 min walk • 5 U-turn test • Static balance test
Cognition	• Information processing speed test (instead of SDMT, includes reaction time)
Mobility via passive monitoring	• Step counts, duration, and asymmetry • high-density activity patterns and high-density mobility patterns
Additional patient characteristics	None

*SDMT* Symbol Digit Modalities Test

We chose to incorporate such novel ambulatory measures in CanProCo as the current methods that we use to measure an MS patient’s clinical status are imperfect, and likely miss a great deal of information. One major problem in current MS clinical practice with respect to measuring disability is that measurements are made only once every 6–12 months, and therefore likely misses individual variation, and that current measures are not sensitive to debilitating symptoms such as depression, anxiety, and fatigue. Moreover, the standard neurological examination cannot accurately quantify components of neurological disability such as fine motor coordination and gait and posture. Mobile apps have the potential to overcome some of these limitations by frequently taking measurements, utilizing novel technologies to measure subtle neurological dysfunction, and include patient-reported outcome measures of mood and cognition.

The ability to better monitor patients from a clinical standpoint, and to more accurately define “clinical progression” in MS is an important exploratory aim of this study. Measures from Floodlight (the parent app of Floodlight Open) have been validated against known measures of global disability in MS in a smaller study [26], and a larger study (Floodlight Open) has launched worldwide. CanProCo presents a unique opportunity to study these novel clinical measures in relation to detailed clinical, neuroimaging, and biological measures, which may allow for the validation of a number of these novel clinical endpoints. Such novel digital measures have great potential to provide significant benefit in evaluation of patients with MS, both scientifically and clinically.

**Conventional and advanced MRI**

Two types of MRI examinations are collected at specified time points. Clinical (i.e. conventional) grade MRIs of the brain that conform to the Consortium of Multiple Sclerosis Centers (CMSC) published clinical protocol guidelines [27] that includes a 3D T1 weighted sequence for brain volume measurement are acquired for all participants, typically annually. A summary of core and optional clinical MRI sequences is listed in Table 10. Research grade MRIs of the brain and cervical spinal cord, with advanced sequences for greater tissue and pathological sensitivity and specificity are administered to sub-cohort and healthy control participants at baseline and follow-up visits 1, 2 and 4. Advanced sequences include measures sensitive to myelin and axonal health as well as detection of the central vein within white-matter lesions, iron rims around white-matter lesions and advanced spinal cord measures. Due to budgetary constraints, research-grade MRIs could not be performed annually, therefore these four time-points were selected as the priority is to evaluate how short-term change in MRI measures predict clinical disability progression in the longer-term. A summary of the research MRI protocol is provided in Table 11.

**Table 10:** Clinical MRI protocol: brain

Parameters	Description
**Field strength**	Scans should be of good quality, with adequate SNR and spatial resolution (in-section pixel resolution of ≤1 × 1 mm))
**Scan prescription**	Use of the sub-callosal plane to prescribe or reformat axial oblique sections
**Coverage**	Whole-brain coverage
**Section thickness and gap**	For 2D = ≤3 mm no gap For 3D = ≤1 mm (≤1.6 mm overcontiguous, reconstructed to ≤1 mm)
**Core sequences**	1. Anatomic 3D inversion recovery–prepared T1W gradient echo 2. Gadolinium single dose, 0.1 mmol/kg given for 30 s*^a^ 3. 3D sagittal T2WI FLAIR^b^ 4. 3D T2WI^b^ 5. 2D axial DWI (≤5-mm sections, no gap)* 6. 3D spoiled gradient echo T1W (non-IR prep) post-gadolinium*^b^
**Optional sequences**	1. Axial proton attenuation 2. Pre- or post-gadolinium axial T1W spin-echo (for chronic black holes) 3. SWI for identification of central vein within T2 lesions
**Notes**	3D series would be typically reconstructed to 3-mm thickness for display and subsequent comparison for lesion counts ^a^ Minimum 5-min delay before obtaining post-gadolinium T1WI. The 3D sagittal FLAIR may be acquired immediately after contrast injection before the 3D FLASH series. ^b^ If unable to perform a 3D acquisition, then perform 2D axial and sagittal FLAIR, axial fast spin-echo T2WI, and axial post-gadolinium T1WI spin-echo at ≤3-mm section thickness with no gap. *Optional

*SNR* Signal-to-Noise Ratio, *2D* 2 Dimensional, *3D* 3 Dimensional, *T2WI* T2-weighted imaging, *FLAIR* Fluid Attenuated Inversion Recovery, *DWI* Diffusion-Weighted Imaging, *IR* Inversion Recovery, *SWI* Susceptibility-Weighted Imaging

**Table 11:** Research MRI protocol: brain and cervical spine

Sequence	Region (acquisition time)	Parameters	Rationale
3D T1W	Brain (~ 6 min)	• Sagittal acquisition • 1x1x1mm or smaller	Volumetric analysis, registration
3D FLAIR	Brain (~ 6 min)	• Sagittal acquisition • 1x1x1mm or smaller • Match geometry to 3D T1W	Lesion identification and quantification. Use with T2*/QSM for central vein, iron rim detection
T2*/QSM	Brain (~ 6 min)	• 3D multi echo gradient echo • Axial acquisition • In plane resolution 0.6 × 0.6 mm • Slice thickness 1 mm (can acquire at 2 mm and reconstruct to 1 mm if available) • TE 5, 13, 20, 27, 34, 41 ms	Identification of iron rims, central veins in lesions, and QSM may help to detect microstructural changes relevant to progression
MWI (optional)	Brain (~ 6 min)	• 3D GRASE • Acquired voxel 1x2x5mm, reconstructed voxel 1x1x2.5 mm • 48 echoes with TE 8 ms	To detect changes in myelin in lesions and NAWM
DTI	Brain (~ 4 min)	• Isotropic voxels 1.7 × 1.7 × 1.7 mm • 30 directions TR/TE = 4700/64 ms • b = 1000s/mm^2^	To use with T2* to assess structural connectivity, ROI definition
MTsat	Brain (~ 9 min)	• MTon and MToff (TR 25 ms, flip angle 5 degrees), T1W (TR 11 ms, flip angle 15 degrees) • TE 3 ms • Off-resonance Gaussian MT pulse • Sagittal 1.5 × 1.5 × 1.5 mm	Detect changes in myelin in lesions and NAWM
PSIR or STIR	Cervical SC (~ 5 min)	• Sagittal 0.7 × 0.7x3mm (or better)	Lesion identification
T2W	Cervical SC (~ 5 min)	• 3D TSE • Sagittal 0.8 × 0.8 × 0.8 mm	Quantifying SC CSA, registration to template, nerve root identification
MTsat	Cervical SC (~ 7–15 min)	• 3D GRE • Axial 0.9 × 0.9x5mm • TE ~ 2 ms • MTon and MToff: TR ~ 50 ms, Flip Angle 9 degrees, T1W: TR = 15 ms, Flip Angle 15 degrees	Quantifying myelin in SC
Multi-slice multi-echo gradient echo	Cervical SC (~ 4 min)	• Resolution should be 0.5 mm in plane • Slice thickness of 5 mm	Lesion identification and grey matter segmentation in SC

*3D* 3 Dimensional, *FLAIR* Fluid Attenuated Inversion Recovery, *QSM* Quantitative Susceptibility Mapping, *TE* Echo Time, *MWI* Myelin Water Imaging, *GRASE* Gradient and Spin Echo, *NAWM* Normal Appearing White Matter, *DTI* Diffusion Tensor Imaging, *TR* Repetition Time, *ROI* Region of Interest, *PD* Proton Density, *MT* Magnetization Transfer, *PSIR* Phase Sensitive Inversion Recovery, *STIR* Short T1 Inversion Recovery, *GE* General Electric, *SC* Spinal Cord, *TSE* Turbo Spin Echo, *CSA* Cross Sectional Area, *MTsat* Magnetization Transfer Saturation, *GRE* Gradient Echo

**Blood and cerebrospinal fluid**

Sub-cohort and healthy control participants give 70 mL of blood at each study visit: 60 mL is collected in Ethylenediaminetetraacetic acid-treated tubes for immediate isolation of peripheral blood mononuclear cells (PBMC) (50 mL) and plasma (10 mL), and 10 mL is collected in a tube with gel for optimal serum collection.

A CSF sample is obtained from consenting sub-cohort participants who are undergoing a lumbar puncture for clinical care, often to confirm a diagnosis of MS. A single additional tube of CSF (approximately 5 mL) is taken for research purposes. CSF is spun down, cell pellet and supernatants are frozen at − 80 °C. This sample can be obtained at any point during study participation, and repeat samples are collected if the participant undergoes another lumbar puncture for clinical reasons and provides consent.

Unbiased, hypothesis-free, single-cell ribonucleic acid (RNA) sequencing analysis of PBMCs of individual participants, collected at the baseline visit will be performed. These data will be used as a “signature peripheral lymphocyte RNA profile”, which will be used to identify signature molecules in PBMCs that are either up-regulated or down-regulated in participants who demonstrate the outcomes of interest (e.g. onset of progression or increased rate of progression). This approach will lead to novel information about MS disease pathways, and will allow linkage of specific molecules, or entire pathways, to clinical outcomes of interest. In addition, a hypothesis-driven evaluation of a wide range of biological and immunological factors underlying progression will be evaluated (Table 12).

**Table 12:** Hypothesis-driven analysis of biological and immune factors that may be associated with progression in MS

Categories of biological and immune factors that may be associated with progression in MS	
• Markers of oxidative stress: plasma levels of lipid hydroperoxides, advanced oxidation protein products, and nitric oxide metabolites • Molecules that signal alterations of the intestinal barrier that can impact on functions of CNS cell types (short chain fatty acids, intestinal fatty acid binding protein, LPS binding protein, LPS in some cases) • Levels of matrix metalloproteinases and inhibitors • Multiplex of cytokines and trophic factors: IL-1β, IL-2, IL-4, IL-5, IL-6, IL-7, IL-8, IL-9, IL-10, IL-11, IL-12, IL-13, IL-15, IL-17, IL-18, IL-21, IL-22, IL-23, IL-26, TNF, BDNF, NGF, leptin and other adipokines, insulin, type I and type II interferons using commercial multiplex platforms. • Multiplex of chemokines: CCL2, CCL11, CXCL1, BAFF, CXCL13, CCL4. • Metabolites using commercial platforms (Metabolon) • Molecules associated with neurodegeneration (Abeta, tau, serum NFL, etc.) • Molecules associated with astrocyte activation (GFAP, CH3L1, etc.) • Profiling of immune cell subsets, cytokine production, and myelin-reactivity of T cell subsets using traditional platforms such as flow cytometry (CyTOF) and ELISPOT • Profiling of autoantibody levels using autoantigen arrays that are spotted with myelin, CNS, and other antigens modulated during oxidative stress • Serum Vitamin D level	

*CNS* Central Nervous System, *IL* Interleukin, *TNF* Tumor Necrosis Factor, *BDNF* Brain Derived Neurotrophic Factor, *NGF* Nerve Growth Factor, *CCL* C Motif Chemokine Ligand, *CXCL* C-X-C Motif Chemokine Ligand, *BAFF* B Cell Activating Factor, *NFL* Neurofilament Light, *GFAP* Glial Fibrillary Acidic Protein, *CH3L* Chitinase-3-Like Protein 1, *CyTOF* Cytometry Time of Flight, *ELISPOT* Enzyme-Linked Immune Absorbent Spot

**Administrative health data linkage**

CanProCo data from all participants residing in British Columbia (BC) and Alberta (AB) will be linked to administrative databases in each province, which will provide valuable information on drug utilization, health care utilization and health care outcomes. Due to the publicly funded health care system that exists across all Canadian provinces, provincial administrative databases capture information on all medically necessary care, including hospitalizations, physician visits, emergency room visits, as well as prescription medications. Tables 13 and 14 summarize the databases used and data collected on CanProCo participants in BC and AB.

**Table 13:** Administrative databases used for linkage to CanProCo data

British Columbia	Alberta
Worksafe British Columbia	Alberta Blue Cross Claims
PharmaNet	Alberta Ambulatory Care Reporting System
Medical Services Plan	Inpatient Discharge Abstract Database
Discharge Abstract Database	Alberta Cancer Registry
Vital Statistics	Communicable Disease Reporting System
British Columbia Multiple Sclerosis Clinical Database	Pharmaceutical Information Network Dispenses
National Ambulatory Care Reporting System	Practitioner Claims Alberta Health Care Insurance Plan
Census	Vital Statistics
Immigration and Refugees Citizenship Canada	Longitudinal Demographic Profile

**Table 14:** Administrative data sought for individuals living with MS in CanProCo site provinces

Variable/Factor	Rationale
Drug utilization	Possible predictor of disease progression
Physician visits	Possible measure of disease severity and thus, potential predictor or indicator of MS progression. Contributor to health care costs and source of comorbidity data.
Hospital admissions	Possible measure of disease severity and thus, potential predictor or indicator of MS progression. Contributor to health care costs and source of comorbidity data.
Emergency room visits	Possible measure of disease severity and thus, potential predictor or indicator of MS progression. Contributor to health care costs and source of comorbidity data.
Vital status	Cohort censoring due to death
LifeLabs	Clinical laboratory data to explore potential predictors of progression

*MS* Multiple Sclerosis

BC and AB were initially selected for administrative data linkages because access to individual provincial administrative databases is a separate costly application. Of the CanProCo site locations, BC and AB are the two provinces with databases that include variables of interest in MS, such as drug utilization and laboratory data. If funding permits, administrative database linkages in Ontario and Quebec will also be explored (i.e.) in the future.

### Outcomes

#### Outcome measure selection

The primary outcome measure in the RIS sub-cohort is development of clinical symptoms leading to a diagnosis of MS, which would include both RRMS and PPMS. Since one of the objectives of CanProCo is to evaluate factors related to the clinical onset of MS, this is the ideal setting to assess for clinical onset of MS as the primary outcome measure of interest. Notably, the onset of clinical symptoms in RIS participants converting to PPMS also enables the evaluation of onset of clinical neurological disability progression, while the onset of clinical symptoms in RIS converting to RRMS enables the evaluation of clinical onset of MS, and therefore evaluates “progression” along the disease spectrum of MS.

The primary outcome measure in the RRMS and PPMS sub-cohorts was chosen to be a composite measure reflecting clinical or radiological change reflective of clinical disease progression in MS. Clinical disease progression based on a meaningful change in EDSS is widely used in clinical trials and in clinical settings to reflect disease progression in MS. We defined meaningful change in EDSS based on widely-utilized clinical trial criteria (outlined above) in “primary outcomes” below. We also included brain atrophy beyond a specific threshold that indicates pathological disease progression as a component of the primary outcome measure, as brain atrophy has been demonstrated to be highly relevant to clinical disability progression in both the short-term and long-term in MS. Numerous clinical trials demonstrate that brain atrophy is highly relevant to disability progression [28], and long-term follow-up studies of clinical trial data have demonstrated the early brain atrophy is highly predictive of EDSS progression over the ensuing 5–10 years [29, 30]. As such, we selected the composite outcome measure of EDSS progression or significant brain volume loss of > 0.94% over 1 year as the primary outcome measure of interest in sub-cohorts 2 and 3. According to previous literature [31], a cut-off denoting a pathological percentage of brain volume change related to MS over 1 year is − 0.4% using the SIENA analysis method. A recent study [32] evaluating measurement variability across various brain volume measurement methods reported that in order to ensure that a patient has a brain volume loss greater than 0.4% over 1 year, the cut-off must be set to − 0.94% (including physiological fluctuations and measurement error).

Secondary outcome measures in the RRMS and PPMS sub-cohorts include disability progression of > 20% in the MSFC measure [24], another global disability scale utilized in MS that captures additional components of disability (upper limb function, cognitive function, and walking speed) that the EDSS may not capture. A change of > 20% has been determined to be a threshold that represents meaningful disability progression in MS. Another secondary outcome measure is deterioration in low-contrast visual acuity, which has been found to be a much more sensitive method to detect pathological changes in visual acuity related to MS [33]. A change in > 7 letters has been found to reflect meaningful clinical deterioration [34].

#### Primary outcomes

##### RIS participants

1. Diagnosis of RRMS (defined by 2017 McDonald criteria) [8] or
2. Diagnosis of PPMS (defined by 2017 McDonald criteria) [8]

##### RRMS participants

1. Disability *progression* defined as: significant EDSS step increase (as per standard clinical trial criteria) or brain atrophy of > 0.94% per year measured using SIENA [32]
2. Rate of *progression* defined as: estimated average change in EDSS (or a transformed version of this variable) per person-year.

Per standard clinical trial criteria a significant EDSS increase represents an increase of > 1.5 if baseline EDSS is between 0 and 1.0; EDSS increase > 1.0 if baseline EDSS between 1.5 and 5.5; EDSS > 0.5 if baseline EDSS > 6.0.

##### PPMS participants

1. Disability *progression* defined as: significant EDSS step increase or brain atrophy of > 0.94% per year measured using SIENA
2. Rate of *progression* defined as: estimated average change in EDSS (or a transformed version of this variable) per year.

#### Secondary outcomes

##### RIS participants converting to PPMS, RRMS and PPMS participants

1. Disability *progression* defined as:
  ▪ > 20% deterioration of modified MSFC (based on MSPT measures) [24]
  ▪ > 20% deterioration of individual modified MSFC components (9 Hole Peg Test, Timed 25 Foot Walk Test, Symbol Digit Modalities Test)
  ▪ Deterioration of low-contrast visual acuity (> 7 letter change) [34]

#### Measuring outcomes

Each participant undergoes a detailed clinical assessment during annual study visits. Information is collected directly by the study coordinator and neurologist (neuro-status certified), as well as through the iPad based MSPT application. This results in detailed clinical and demographic information, as well as completion of the components of the MSFC. Participants who have enrolled in the Floodlight Open digital application provides the CanProCo site with their unique identifier, so their digital data can be downloaded from the Floodlight Open web-based database. All participants will also have a clinical and/or research-grade MRI from which annual rates of brain atrophy will be calculated.

The primary outcome in the RIS sub-cohort will be determined by assessing if the participant reports typical symptoms suggestive of a clinical relapse or progressive neurological symptoms. This will be confirmed by performing a full neurological examination. If there is evidence of new clinical disability, which improves over time, this will support RIS developing a diagnosis of clinically isolated syndrome or RRMS. Moreover, if there is evidence of disability progression, this will further support RIS developing a diagnosis of PPMS.

For RRMS and PPMS participants, a composite measure (clinical progression, as defined by the EDSS typically used in clinical trial settings) or greater than 0.94% per year rate of change of brain atrophy will be utilized. Each participant is evaluated at annual study visits by a neurostatus-experience study neurologist to determine if there has been EDSS progression.

Secondary outcomes will be determined using the MSFC and visual acuity. Exploratory outcomes, including combinations of various secondary clinical outcome measures will be also evaluated in the context of other known measures, such as EDSS and the MSFC.

Information regarding whether there has been a relapse within 90 days of the study visit is collected routinely as part of each study visit. If the study visit is taking place within 90 days of a clinical relapse, whenever possible, the study participant is brought in for a “relapse recovery” visit at least 90 days later, when clinical outcomes are collected as a new neurological baseline. Since a recent relapse will affect primary and secondary outcome measures, any subsequent analyses will take into account whether a relapse took place in close temporal proximity to the study visit, and will incorporate “relapse recovery” clinical information, as appropriate.

### Study status

The first CanProCo participant was enrolled in April 2019 and recruitment is on-going. Study activities were paused for a minimum of 6 months across all sites in mid-March 2020 due to the coronavirus-19 pandemic, and there has been subsequent study re-opening, followed by additional pauses as the pandemic continues. Due to these delays, recruitment is expected to continue for another year, into mid-2022.

#### Sample size justification and power calculations

##### Sample size justification for foundation cohort

The sample size of the foundation cohort (*n* = 1000) was based on pragmatic logistical and budgetary constraints. Specifically, the number of study sites (*n* = 5) and the number of MS participants that can realistically be seen on an annual (or more frequent) basis at each study site (*n* = 200) determined the sample size.

However, given that participants included will conform to relatively restrictive criteria (RRMS, PPMS, RIS) within 10–15 years of disease onset, this sample size (*n* = 1000) is likely adequate to detect clinical, epidemiological, and health systems factors with moderate effect sizes on the outcomes of interest in this study (de novo onset of disease progression, and rate of disease progression). This statement is supported by the fact that many prospective cohorts around the world that have produced adequately precise estimates of epidemiological parameters in MS have had sample sizes of approximately 500–2000. These include the University of California San Francisco EPIC [35] cohort (*n* = 500), the Barcelona CIS [36] cohort (*n* = 900, based on recent 10-year follow-up study), the Harvard CLIMB [37] cohort (*n* = 2000), the SUMMIT [38] cohort (*n* = 1000), the Winnipeg MS cohort (*n* = 2000), the Swiss MS [39] cohort (*n* = 900), the German prospective MS [40] cohort (*n* = 1000), and a proposed Australian prospective MS cohort (proposed *n* = 1500) [41].

##### Sample size justification for sub-cohorts

A summary table of the odds ratios, which represents the difference in effect of a continuous or binary micro/macro factor in those that develop the outcome of interest (vs. not) in each sub-cohort is provided in Table 15 below. Summary paragraphs for each sub-cohort are provided below and additional details are provided in Additional file 1.

**Table 15:** Sample size justification for sub-cohorts

Sub-cohort	Proposed sample size	Effect size for continuous variable (OR)	Effect size for binary variable (OR)	Power (1 – Beta)
**RIS**	*n* = 100	1.8	3.7	0.80
**RIS**	*n* = 100	2.0	4.8	0.90
**RIS**	*n* = 150	1.6	2.8	0.80
**RIS**	*n* = 150	1.8	3.4	0.90
**RRMS**	*n* = 200	0.6	0.3	0.80
**RRMS**	*n* = 200	0.6	0.4	0.90
**PPMS**	*n* = 100	0.5	0.2	0.80
**PPMS**	*n* = 100	0.5	0.2	0.90

*OR* Odds Ratio, *RIS* Radiologically Isolated Syndrome, *RRMS* Relapsing Remitting Multiple Sclerosis, *PPMS* Primary Progressive Multiple Sclerosis

##### Sub-cohort 1: RIS (*n* = 100 and *n* = 150)

**Outcome of interest: Diagnosis of MS (RRMS or PPMS)**

Summary: Odds ratio, which represents the difference in effect between those that develop MS vs. not) is 1.6–1.8 for a continuous variable, and 2.8–3.4 for a binary variable, which is reasonable for most advanced neuroimaging and biological measures.

##### Sub-cohort 2: RRMS within 5 years of diagnosis, treatment naïve (or not on disease modifying therapy > 12 months)

Outcome of interest: disease “progression” (as defined by EDSS according to typical clinical trial criteria or brain atrophy rate). Summary: Odds ratio (difference in effect between RRMS that develop progression vs. those RRMS that do not) is 0.59–0.64 for a continuous variable, 0.3–0.36 for a binary variable, which is reasonable for most imaging and biological measures.

##### Sub-cohort 3: PPMS within 10 years of onset (*n* = 100)

Outcome of interest: disease “progression” (as defined by EDSS according to typical clinical trial criteria or brain atrophy rate).

Summary: Odds ratio (difference in effect between PPMS that develop progression vs. PPMS that do not) is 0.49–0.54 for a continuous variable, 0.17–0.23 for a binary variable, which is reasonable for most imaging and biological measures.

## Standardization and data quality

Over the course of a 10 month feasibility and planning grant (July 2017 – May 2018), several SOPs were developed and refined for each CanProCo scientific pillar. During this period, required infrastructure (e.g. study databases) to securely obtain, process, and store data spanning all pillars were assessed, optimized, and finalized.

### Neuro-immunology pillar

A number of test dry runs were performed at the Montreal CanProCo site (Centre hospitalier de l’Université de Montréal) to ensure pillar aims and SOPs achieved proposed goals. Among these, PBMCs from eight healthy control volunteers were processed using different available procedures/kits. Cell fractionation was performed in parallel by two experienced technical staff to obtain cluster of differentiation (CD)19, CD4, CD8, and CD14 leukocytes. The decision was made to pursue single cell RNA sequencing technology because none of the examined kits conferred more than 95% cell purity. Proof of feasibility for use of single cell RNA sequencing was confirmed by favourable RNA yield obtained from PBMCs frozen for different periods of time [42–44].

An in-person training session was held in Montreal prior to study launch, attended by laboratory technician staff from all CanProCo sites. Each technician was trained on the SOPs for plasma and serum processing, and PBMC isolation. Based on this training, additional changes were made to optimize the SOPs. Each CanProCo site then performed its own (local) dry run using healthy control volunteer blood and samples were shipped to Montreal for quality assurance analysis, and to ensure the shipping protocol was adequate for all sites and their shipping locations.

### Neuro-imaging pillar

Questionnaires were sent to all potential CanProCo sites that met initial eligibility criteria (defined above) to better understand each site’s clinical and research imaging capacity. Each site submitted information on its current MRI platform (make, model, magnet strength) and current clinical MS MRI protocol sequences, which were assessed and reviewed by the neuroimaging pillar leads. Feedback was sent to each site to determine whether existing protocols could be standardized to administer sequences according to published CMSC guidelines [27].

Feasibility assessment activities for research grade (i.e. advanced) MRI consisted primarily of formulating and circulating questionnaires to confirm interest and capacity to participate in advanced MRIs for CanProCo sub-cohort participants. This was done after the assessment of clinical MRI protocols for each site. Sites with displayed experience, interest, and ability to collect research-grade scans were provided with detailed SOPs developed by the neuroimaging pillar leads for brain and spinal cord data collection.

After the final selection of participating CanProCo sites, test scans were performed using the advanced MRI protocol at each centre and sent to the University of British Columbia for review and quality assurance. After study launch, the first three clinical and research-grade MRI scans captured at each CanProCo site were sent to the University of British Columbia for additional review and, where applicable, supplemental SOP changes were made based on site-specific needs.

### Epidemiology and health outcomes pillar

After final decisions were made regarding case report form data and number and types of questionnaires administered to CanProCo participants, several electronic storage platforms were evaluated to potentially house these data. The decision was made to partner with the Praxis Spinal Cord Institute (formerly the Rick Hansen Institute) who developed a customized CanProCo electronic database using their GRP. Multiple rounds of review were made to the trial version of the database to ensure alignment with local screening and enrolment logs, paper case report forms, and specifically to ensure correct algorithms were programmed into GRP to reflect order of question presentation for questionnaires with response driven instructions. Comprehensive SOPs were developed to ensure research coordinators have clear guidance on how to enter all types of study data. This serves as a robust training tool for new study staff, and also ensures updated/revised versions of case report forms and questionnaires remain compatible with the GRP interface. Before real-time deployment of the GRP database, each site coordinator entered 10 visits’ worth of data and the data export was reviewed for data quality (i.e. outlying values) and missing data. Monthly audits of the data are performed by the CanProCo study manager, and any outlying values or significant amounts of missing data flagged and investigated further. Any changes to database data are logged in GRP for record keeping. Ongoing training occurs during monthly site coordinator teleconference calls to ensure continued data accountability and quality.

Health administrative database access requests were made in the provinces of BC and AB. Once data access is granted, data cleaning and harmonization across provinces, as well as selection of case definitions will take place prior to data analysis.

## Statistical analyses

Descriptive analyses of baseline clinical, demographic, MRI measures, and biological measures will be provided for the entire cohort, and by disease phenotype. Longitudinal changes in these measures will be provided. Continuous variables will be summarized using mean/median and standard deviation or inter-quartile range, and categorical variables will be summarized using proportions. Standardized protocols, forms, and databases are utilized for data collection to minimize sources of bias.

Retention will be evaluated annually, and attempts made to increase study retention if problems are identified. If one particular cohort has a higher rate of drop-out or loss to follow-up, subsequent statistical analyses will have to take missing data into account, and results will need to be interpreted with this limitation in mind. Multiple imputation may be considered if missing data are substantial and follow a missing at random pattern.

Differences between disease phenotypes in baseline characteristics will be evaluated using ANOVA or chi-squared tests, depending on whether the variable is continuous or categorical.

Multivariable logistic regression and Cox models will be utilized to evaluate relationships between the primary outcome measure in relation to an independent variable (biological, imaging, clinical, epidemiological) factor of interest. In addressing objectives concerned with rates of progression, models for repeated measures (e.g. GEE, mixed models) will be used and time by covariate interaction terms will be used to identify determinants of progression rates. To explore the integrative aim of characterizing the interactions and associations between factors in different scientific pillars associated with de novo onset *and rate* of progression in MS, factors from different scientific pillars will be included in specific models, and their relative contributions to onset and rate of progression, as well as interactions between these factors will be evaluated. In each logistic regression model, relevant confounding variables will be included. If there are two independent variables where effect modification is suspected, an interaction variable including these two covariates will be included in the model. Sensitivity analyses will be performed as needed in exploratory analyses to confirm or refute preliminary or equivocal findings. Exploratory analyses related to prediction will use statistical learning or machine learning techniques for model development and will use unseen data for validation of prediction models.

## Scientific advisory board

An independent scientific advisory board (SAB) comprised of clinicians and scientists with content expertise and experience in scientific fields relevant to CanProCo was assembled in September 2018. CanProCo holds formal annual meetings with the SAB to update them on progress, and to solicit feedback and advice on any key issues related to study conduct, funding, governance, and analysis. In between annual meetings, CanProCo periodically solicits feedback from the SAB when necessary. While CanProCo solicits and considers carefully feedback from the SAB, the CanProCo Executive Board Committee retains the right to come to major study decisions independently. Current members of CanProCo’s SAB include: Amit Bar-Or (USA), Helmut Butzkueven (Australia), Daniel Reich (USA), Alan Thompson (UK), Heinz Wiendl (Germany), and Christina Wolfson (Canada).

## Discussion

This study is highly relevant to the lives of people with MS across the entire spectrum of disease, as MS is a disease continuum, and progression is a component of MS from disease onset. A better understanding of the myriad micro- and macro- factors related to progression in MS, and how they interact to cause progression in MS will build the necessary foundation to developing better disease-modifying treatments and treatment strategies that improve the lives of people living with all types of MS, but especially those where progression is the prevalent clinical feature. This is truly the greatest unmet need in the clinical practice of MS, as exemplified by the fact that despite decades of experimental therapeutics, treatments that truly alter the trajectory of progressive disease do not yet exist.

The proposed study design of CanProCo is innovative and a national cohort study focused specifically on understanding progression in MS is unprecedented. Moreover, the intended single cell RNA sequencing analyses of leukocyte populations and relationship to progression will offer an unbiased assessment of the contribution of the immune system, and mechanisms thereof, to clinically meaningful events in MS. CanProCo builds upon many strengths that exist within Canada, which include: the high prevalence of MS, centralized MS care across provinces, and the availability of administrative databases which will allow an assessment of health systems factors relevant to progression in MS. Moreover, the existence of expertise across a wide range of scientific pillars and investigators that have long-standing productive collaborations is another strength that bodes well for the productivity and longevity of the study.

The depth and breadth of data collection in CanProCo across diverse fields will enable an assessment of progression in MS that individual isolated studies do not have the capacity or resources to evaluate. Over time, this rich dataset will enhance our understanding of MS disease processes, and will also have direct translational potential by facilitating the development of improved, personalized treatment strategies.

CanProCo is also a unique example of multiple funding sources coming together to accomplish a collective goal. Specifically, CanProCo has brought together non-profit organizations (MS Society of Canada), federal funding organizations (Brain Canada), provincial funders (Government of Alberta), and industry partners (Biogen-Idec and Roche) to work towards a collective goal in the field.

Finally, in an attempt to foster collaborations and improve efficiency in the MS field, CanProCo’s study design (inclusion/exclusion criteria, selection of outcome measures) and data collection methods took into account existing studies which utilize similar methodologies, to increase the possibility of future collaborative studies [41]. Moreover, once a critical amount of data are collected, the eventual goal will be to make CanProCo data accessible to qualified investigators around the world.

For all of these reasons, CanProCo is positioned to make a unique and substantial contribution to the field. Together with other ongoing international efforts, results from CanProCo will allow the field to advance closer to preventing progression in MS, ultimately improving the lives of people living with MS.

## Supplementary Information

**Additional file 1.** Sample size justification for sub-cohorts. Description: Sample size justifications for sub-cohorts, prepared by Dr. Maria Pia Sormani.

## Data Availability

At completion of the study, all participating sites will have access to final datasets. After study completion, study data will be made available to qualified external investigators through a data access request and review process. Results from the study will be communicated through abstracts at scientific meetings, peer-reviewed publications, and educational events held in conjunction with the MS Society of Canada.

## Abbreviations

MS: Multiple sclerosis
RRMS: Relapsing remitting multiple sclerosis
PPMS: Primary progressive multiple sclerosis
CanProCo: Canadian Prospective Cohort Study to Understand Progression in Multiple Sclerosis
RIS: Radiologically isolated syndrome
MRI: Magnetic resonance imaging
SOP: Standard operating procedure
CSF: Cerebrospinal fluid
GRP: Global research platform
EDSS: Expanded disability status scale
MSPT: Multiple Sclerosis Performance Test
MSFC: Multiple Sclerosis Function Composite
CMSC: Consortium of Multiple Sclerosis Centers
PBMC: Peripheral blood mononuclear cells
RNA: Ribonucleic acid
BC: British Columbia
AB: Alberta
CD: Cluster of differentiation
SAB: Scientific Advisory Board
