# Supplementary material for: The Canadian prospective cohort study to understand progression in multiple sclerosis (CanProCo): rationale, aims, and study design
Source: BMC Neurol. 2021 Oct 27;21:418. doi: 10.1186/s12883-021-02447-7 (PMC8549411; doi:10.1186/s12883-021-02447-7)
Supplement: Supplementary file 1 — Additional file 1. Sample size justification for sub-cohorts. Description: Sample size justifications for sub-cohorts, prepared by Dr. Maria Pia Sormani. [file 12883_2021_2447_MOESM1_ESM.pdf]

SAMPLE SIZE JUSTIFICATION FOR SUBCOHORTS

Prepared by Dr. Maria Pia Sormani

Subcohort 1: RIS with spinal cord lesions (n=100 and n=150)

Outcome of interest: Conversion to MS (RRMS or PPMS)
Summary: Odds ratio (OR), which represents the difference in effect between those that develop MS vs. not) is 1.6-1.8 for a continuous variable, and 2.8-3.4 for a binary variable, which is reasonable for most advanced neuroimaging and biological measures.

Scenario with n=100 RIS

To assess a "micro" or "macro" factor of interest that is a continuous variable, we will estimate the odds ratio (OR) that can be detected when increasing the baseline factor level of 1 standard deviation above the mean value. An adjustment was made assuming that a multiple regression of the independent variable of interest on the other independent variables in the logistic regression to have a correlation of 0.4 (an R-squared of 0.16).

Logistic Regression Power Analysis

Numeric Results

Power	N	P0	P1	Odds Ratio	R Squared	Alpha	Beta
0.90	100	0.50	0.67	2.03	0.16	0.05	0.10
0.80	100	0.50	0.65	1.84	0.16	0.05	0.20

Report Definitions
Power is the probability of rejecting a false null hypothesis. Here it has been set to 80% or 90%.
N is the size of the sample drawn from the population, here set to 100.
P0 is the probability of conversion to MS at the mean of X, where X is the continuous variable to be studied (baseline biomarker). Here set to 50% (at 5 years).
P1 is the probability of conversion to MS when X is increased to one standard deviation above the mean.
Odds Ratio is the odds ratio when P1 is in the numerator. That is, it is [P1/(1-P1)]/[P0/(1-P0)].
R-Squared is the R2 achieved when X is regressed on the other independent variables in the regression.
Alpha is the probability of rejecting a true null hypothesis.
Beta is the probability of accepting a false null hypothesis.

Summary Statements
A logistic regression of a binary response variable (conversion to MS) on a continuous, normally distributed variable (X) with a sample size of 100 observations achieves 90% power at a 0.05 significance level to detect a change in the probability of conversion to MS after 5 years from the value of 50% at the mean of X to 67% when X is increased to one standard deviation above the mean. This change corresponds to an odds ratio of 2. An adjustment was made assumed that the variable of interest is correlated to other variables included in the model with a global R squared of 0.16. If we accept a power of 80%, the OR that we will be able to detect is 1.84.

Using the same assumptions, for a binary biomarker, the calculation are as follows:

Logistic Regression Power Analysis

Numeric Results

Power	N	Pcnt N X=1	P0	P1	Odds Ratio	R Squared	Alpha	Beta
0.90	100	50,000	0.50	0.83	4.75	0.16	0.05	0.10
0.80	100	50,000	0.50	0.79	3.73	0.16	0.05	0.20

A logistic regression of a binary response variable (conversion to MS) on a binary independent variable (X) with a sample size of 100 observations (of which 50% are in the group X=0 and 50% are in the group X=1) achieves 90% power at a 0.05 significance level to detect a change in in the probability of conversion to MS after 5 years from the value of 50% to 83%. This change corresponds to an odds ratio of 4.75. For an 80% power the detectable OR=3.73.

<u>**Scenario with n=150 RIS**</u>

Continuous biomarker

Logistic Regression Power Analysis

Numeric Results

Power	N	P0	P1	Odds Ratio	R Squared	Alpha	Beta
0.90	150	0.50	0.64	1.78	0.16	0.05	0.10
0.80	150	0.50	0.62	1.65	0.16	0.05	0.20

Binary biomarker

Logistic Regression Power Analysis

Numeric Results

Power	N	Pcnt N X=1	P0	P1	Odds Ratio	R Squared	Alpha	Beta
0.90	150	50,000	0.50	0.77	3.41	0.16	0.05	0.10
0.80	150	50,000	0.50	0.74	2.85	0.16	0.05	0.20

<u>**Subcohort 2: RRMS within 5 years of diagnosis, treatment naïve (or not on DMT > 12 months) with > 100 "high" disease activity**</u>

Outcome of interest: disease "progression" (as defined by EDSS according to typical clinical trial criteria OR brain atrophy rate).

SUMMARY: OR (difference in effect between RRMS that develop progression vs those RRMS that do not) is 0.59-0.64 for a continuous variable, 0.3-0.36 for a binary variable, which is reasonable for most imaging and biological measures.

For this sample size estimation, placebo arms of Phase III clinical trials, that include patients selected for activity according to the mentioned criteria and that were treatment naïve.

According to the previous literature (De Stefano et al, JNNP 2014) a cut-off denoting a pathological percentage brain volume change (PBVC) over 1 year is -0.4% using the SIENA analysis method. According to a paper recently published by Opfer et al (Journal of Neurology 2018) in order to be sure that a patient has a brain volume loss higher than 0.4% over 1 year, the cutoff must be set to -0.94% (including physiological fluctuations and measurement error).

The outcome is therefore set a disability progression or a pathological brain volume loss over 1 year. Data from placebo arms of clinical trials indicate that the percentage of patients with a disability progression or a PBVC higher than -0.94% are around 35% (data on file).

The first case is for assessing a factor represented by a continuous variable, and we estimate the OR we can detect when increasing the baseline factor level of 1 standard deviation above the mean value. An adjustment was made assuming that a multiple regression of the independent variable of interest on the other independent variables in the logistic regression to have a correlation of 0.4 (an R-squared of 0.16).

Logistic Regression Power Analysis – Biomarker as a continuous variable

Numeric Results

Power	N	P0	P1	Odds Ratio	R Squared	Alpha	Beta
0.90	200	0.35	0.24	0.59	0.16	0.05	0.10
0.80	200	0.35	0.26	0.64	0.16	0.05	0.20

Summary Statements
A logistic regression of progression over 1 year (defined as an EDSS progression event or a PBVC<-0.94%) on a continuous, normally distributed variable (X) with a sample size of 200 observations achieves 90% power at a 0.05 significance level to detect a change in the risk of progression from the value of 35% at the mean of X to the value of 24% when X is increased to one standard deviation above the mean. This change corresponds to an odds ratio of 0.59. For a power of 80% the OR is 0.63.

Logistic Regression Power Analysis – Biomarker as a binary variable

Numeric Results

Power	N	Pcnt N X=1	P0	P1	Odds Ratio	R Squared	Alpha	Beta
0.90	200	50,000	0.35	0.14	0.30	0.160	0.05	0.10
0.80	200	50,000	0.35	0.16	0.36	0.160	0.05	0.20

Summary Statements
A logistic regression of progression over 1 year (defined as an EDSS progression event or a PBVC<-0.94%) on a binary independent variable (X) with a sample size of 200 observations (of which 50% are in the group X=0 and 50% are in the group X=1) achieves 90% power at a 0.05

significance level to detect a change in the risk of progression from the baseline value of 35% to 13.8%. This change corresponds to an odds ratio of 0.297. For a power of 80% the detectable OR is 0.36 (Hsieh, Bloch & Larsen, Stat Med 1998).

<u>Subcohort 3: PPMS within 5 years of onset (n=100)</u>

Outcome of interest: disease "progression" (as defined by EDSS according to typical clinical trial criteria OR brain atrophy rate).

SUMMARY: OR (difference in effect between PPMS that develop progression vs. PPMS that do not) is 0.49-0.54 for a continuous variable, 0.17-0.23 for a binary variable, which is reasonable for most imaging and biological measures.

For this sample size estimation data from a PPMS clinical trial were utilized, selecting patients with less than 5 years of disease duration (Sormani, personal data on file).

The outcome is therefore set a disability progression or a pathological brain volume loss over 1 year. Data from the PPMS trial indicate that the percentage of patients with a disability progression or a PBVC higher than -0.94% are around 40% (data on file).

The first case is for assessing a factor represented by a continuous variable, and we estimate the OR we can detect when increasing the baseline factor level of 1 standard deviation above the mean value. An adjustment was made assuming that a multiple regression of the independent variable of interest on the other independent variables in the logistic regression to have a correlation of 0.4 (an R-squared of 0.16).

Logistic Regression Power Analysis

Numeric Results4

Power	N	P0	P1	Odds Ratio	R Squared	Alpha	Beta
0.90	100	0.40	0.25	0.49	0.16	0.05	0.10
0.80	100	0.40	0.26	0.54	0.16	0.05	0.20

Summary Statements

A logistic regression of progression over 1 year (defined as an EDSS progression event or a PBVC<-0.94%) on a continuous, normally distributed variable (X) with a sample size of 100 observations achieves 90% power at a 0.05 significance level to detect a change in the risk of progression from the value of 40% at the mean of X to the value of 24.5% when X is increased to one standard deviation above the mean. This change corresponds to an odds ratio of 0.49. For a power of 80% the OR is 0.54.

Logistic Regression Power Analysis

Numeric Results

Power	N	Pcnt N X=1	P0	P1	Odds Ratio	R Squared	Alpha	Beta
0.90	100	50,000	0.40	0.10	0.168	0.16	0.05	0.10
0.80	100	50,000	0.40	0.13	0.231	0.16	0.05	0.20

Summary Statements

A logistic regression of progression over 1 year (defined as an EDSS progression event or a PBVC<-0.94%) on a binary independent variable (X) with a sample size of 100 observations (of which 50% are in the group X=0 and 50% are in the group X=1) achieves 90% power at a 0.05 significance level to detect a change in the risk of progression from the baseline value of 40% to 10%. This change corresponds to an odds ratio of 0.17. For a power of 80% the detectable OR is 0.23.
